# Supplementary material for: Hybrid Spreading Mechanisms and T Cell Activation Shape the Dynamics of HIV-1 Infection
Source: PLoS Comput Biol. 2015 Apr 2;11(4):e1004179. doi: 10.1371/journal.pcbi.1004179 (PMC4383537; doi:10.1371/journal.pcbi.1004179)
Supplement: S1 Table — Results are shown for all 17 patients. In the data, time is recorded relative to the first appearance of symptoms of HIV infection. As the actual initial infection date is unknown, we assumed a constant “eclipse” phase of 20 days between initial infection and first appearance of symptoms. (PDF) [file pcbi.1004179.s001.pdf]

Patient	$Q_0\,(cells/\mu l)$	$S_0\,(cells/\mu l)$	$N_M\,(cells/\mu l)$	$\kappa\,(day^{-1})$	$D(days)$
MM1	879	21	996	1.753604	30
MM4	684	16	773	1.474027	33
MM8	392	9	439	1.076508	49
MM9	440	10	494	1.349088	61
MM12	542	13	611	1.982885	72
MM13	537	13	605	1.250311	31
MM23	419	10	470	1.150800	51
MM24	547	13	617	1.494605	54
MM27	606	14	684	1.353939	30
MM33	781	18	884	1.623077	33
MM39	553	13	623	1.276949	32
MM40	435	10	488	1.137114	46
MM42	641	15	724	1.801382	57
MM43	582	14	656	2.511129	80
MM45	542	13	611	1.273904	33
MM57	406	9	455	1.207627	58
MM60	587	14	662	1.325221	33
