# Supplementary material for: Hybrid Spreading Mechanisms and T Cell Activation Shape the Dynamics of HIV-1 Infection
Source: PLoS Comput Biol. 2015 Apr 2;11(4):e1004179. doi: 10.1371/journal.pcbi.1004179 (PMC4383537; doi:10.1371/journal.pcbi.1004179)
Supplement: S2 Table — N S is the quasi-steady CD4+ T cell density and V S is the quasi-steady density of free virions, which are average densities between the 100th and 800th days after initial infection. t A is the time to AIDS, which is defined as the time between initial infection and when the density of CD4+ T cells falls to 200 cells/μl. (PDF) [file pcbi.1004179.s002.pdf]

Patient	$N_s\,(cells/\mu l)$		$log_{10}V_s\,(virions/\mu l)$		$t_A\,(days)$	
	Data	Model	Data	Model	Data	Model
MM1	851	829	1.63	1.45	>3317	3342
MM4	663	646	1.11	1.38	>1567	1612
MM8	367	354	1.36	1.41	846	978
MM9	368	359	1.64	1.64	895	1101
MM12	386	377	1.84	1.86	935	931
MM13	545	510	0.90	1.30	>1151	1162
MM23	381	374	1.68	1.45	1554	1574
MM24	462	453	1.72	1.69	>1093	1152
MM27	610	579	1.15	1.30	1389	1389
MM33	745	729	1.51	1.46	>2051	2061
MM39	558	526	1.08	1.30	1226	1286
MM40	408	400	1.26	1.36	>1550	1572
MM42	508	499	1.78	1.81	>966	1022
MM43	367	347	2.12	1.98	>787	786
MM45	543	521	0.30	1.23	>3068	3217
MM57	352	346	1.52	1.56	>1240	1387
MM60	582	554	0.60	1.36	>1140	1149
