# Supplementary figures and images for: Hybrid Spreading Mechanisms and T Cell Activation Shape the Dynamics of HIV-1 Infection
Source: PLoS Comput Biol. 2015 Apr 2;11(4):e1004179. doi: 10.1371/journal.pcbi.1004179 (PMC4383537; doi:10.1371/journal.pcbi.1004179)

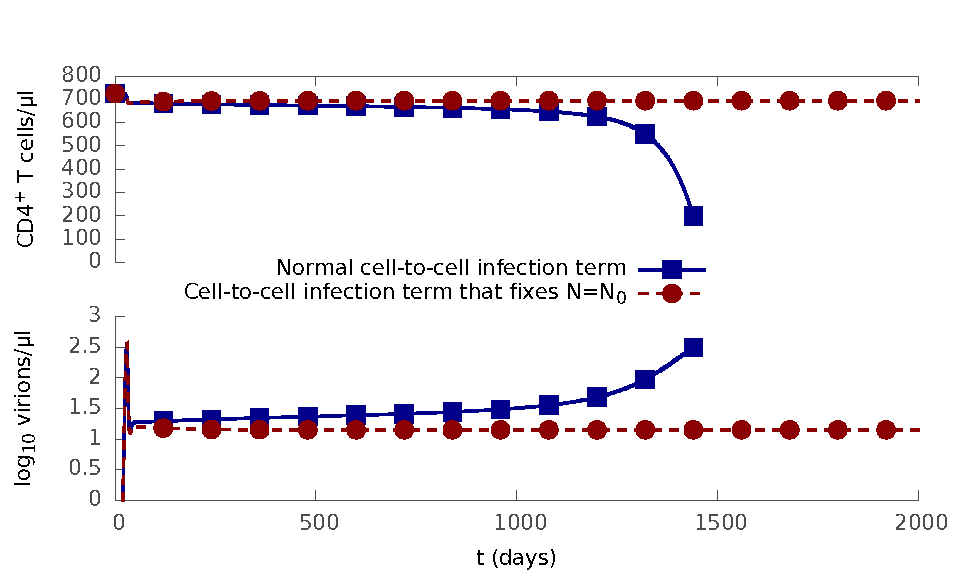

Supplement: S1 Fig — 1) the normal cell-to-cell infection term (cθβ 1 IS/N) in Equation 1, and 2) the cell-to-cell infection term that fixes N to be N 0 (cθβ 1 IS/N 0 where N 0 is the total number of CD4+ T cells before HIV-1 infection and it is a constant). With a fixed N = N 0 in the term, the HIV-1 model would not be able to recapitulate the AIDS phase. (TIFF) [file pcbi.1004179.s003.tiff]

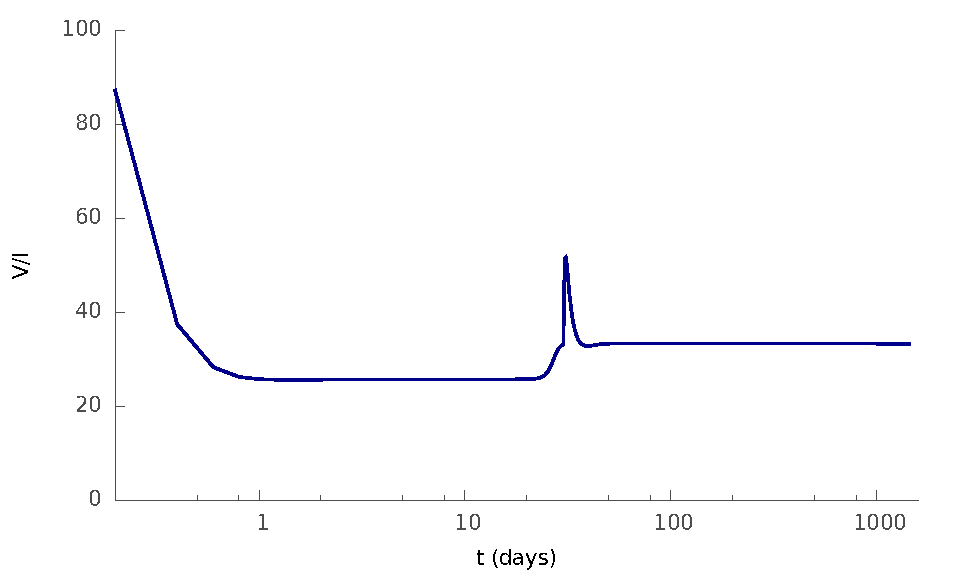

Supplement: S2 Fig — There are some obvious changes in V/I during the early days of HIV-1 infection. The figure however also supports some existing works’ [8] assumption that V and I are proportional to each other during the chronic (stable) phrase of HIV-1 infection. Our model depicts HIV-1 infection in all its three phrases and we do not therefore assume V to be proportional to I. (TIFF) [file pcbi.1004179.s004.tiff]
